# Advanced Imaging Analysis in Prostate MRI: Building a Radiomic Signature to Predict Tumor Aggressiveness

**DOI:** 10.3390/diagnostics11040594

**Published:** 2021-03-26

**Authors:** Anna Damascelli, Francesca Gallivanone, Giulia Cristel, Claudia Cava, Matteo Interlenghi, Antonio Esposito, Giorgio Brembilla, Alberto Briganti, Francesco Montorsi, Isabella Castiglioni, Francesco De Cobelli

**Affiliations:** 1 Department of Radiology, IRCCS San Raffaele Scientific Institute, 20132 Milan, Italy; damascelli.anna@hsr.it (A.D.); cristel.giulia@hsr.it (G.C.); esposito.antonio@hsr.it (A.E.); brembilla.giorgio@hsr.it (G.B.); decobelli.francesco@hsr.it (F.D.C.); 2 Institute of Molecular Bioimaging and Physiology, National Research Council (IBFM-CNR), 20090 Segrate, Italy; francesca.gallivanone@ibfm.cnr.it (F.G.); claudia.cava@ibfm.cnr.it (C.C.); matteo.interlenghi@ibfm.cnr.it (M.I.); 3 Vita-Salute San Raffaele University, 20132 Milan, Italy; briganti.alberto@hsr.it (A.B.); montorsi.francesco@hsr.it (F.M.); 4 Department of Urology, IRCCS San Raffaele Scientific Institute, 20132 Milan, Italy; 5 Department of Physics “G. Occhialini”, University of Milano, 20126 Bicocca, Italy

**Keywords:** magnetic resonance imaging, prostate cancer, prostate cancer aggressiveness, radiomics

## Abstract

Radiomics allows the extraction quantitative features from imaging, as imaging biomarkers of disease. The objective of this exploratory study is to implement a reproducible radiomic-pipeline for the extraction of a magnetic resonance imaging (MRI) signature for prostate cancer (PCa) aggressiveness. One hundred and two consecutive patients performing preoperative prostate multiparametric magnetic resonance imaging (mpMRI) and radical prostatectomy were enrolled. Multiparametric images, including T2-weighted (T2w), diffusion-weighted and dynamic contrast-enhanced images, were acquired at 1.5 T. Ninety-three imaging features (Ifs) were extracted from segmentation of index lesion. Ifs were ranked based on a stability rank and redundant Ifs were excluded. Using unsupervised hierarchical clustering, patients were grouped on the basis of similar radiomic patterns, whose association with Gleason Grade Group (GGG), extracapsular extension (ECE), and nodal involvement (pN) was tested. Signatures composed by IFs from T2w-images and Apparent Diffusion Coefficient (ADC) maps were tested for the prediction of GGG, ECE, and pN. T2w radiomic pattern was associated with pN, ECE, and GGG (*p* = 0.027, 0.05, 0.03) and ADC radiomic pattern was associated with GGG (*p* = 0.004). The best performance was reached by the signature combing IFs from multiparametric images (0.88, 0.89, and 0.84 accuracy for GGG, pN, and ECE). A reliable multiparametric MRI radiomic signature was extracted, potentially able to predict PCa aggressiveness, to be further validated on an independent sample.

## 1. Introduction

Prostate cancer (PCa) is a heterogeneous disease characterized by a wide spectrum of clinical presentations and possible outcomes [1]. In clinical practice, the main challenge is to identify the best balance between limiting overtreatment and reducing mortality.

In the preoperative setting, PCa aggressiveness is commonly established using Gleason grade group (GGG) derived from prostate biopsy specimens that, in 30–50% of cases, does not represent the true GGG of the tumor [2,3,4]. The identification of a non-invasive and accurate tool to predict tumor aggressiveness, taking into account the biological heterogeneity present in whole tumor volume, is an important unmet need.

In the last decade, Radiomics emerged as a post-processing imaging analysis that allows the extraction of large number of quantitative features that cannot be studied solely by visual assessment [5,6]. The role of radiomic in oncological setting is to identify reliable biomarkers of cancer aggressiveness [7,8]. In recent years, several studies explored a radiomic approach applied to multiparametric magnetic resonance imaging (mpMRI) for detection and characterization of PCa [8,9]. Despite the recommendations of international working groups to develop a standardized pipeline to extract image biomarkers, they are characterized by a great variability in methodological approach and results. Furthermore, most of these studies focused the analysis on the prediction of GGG solely, while the association of radiomic features with other important prognostic parameters, such as extracapsular extension (ECE) and nodal involvement, remains quite unexplored.

This study aimed to implement a reproducible radiomic-pipeline for the extraction of a radiomic signature from mpMRI T2w images and Apparent Diffusion Coefficient (ADC) maps able to predict Pca aggressiveness, in terms of GGG, ECE, and nodal stage (pN).

## 2. Materials and Methods

This is a retrospective study approved by our Institutional Review Board; written informed consent was obtained from all patients.

### 2.1. Study Population

The study cohort consisted of 102 men with biopsy proven PCa who underwent both preoperative mpMRI of the prostate and radical prostatectomy at a single tertiary care referral center, between January 2016 and March 2019.

Pathological data of surgical specimen included Gleason grade group (GGG), extracapsular extension (ECE), and nodal stage (pN).

Prostate specimens were processed according to the Stanford protocol [10] and analyzed by a dedicated uro-pathologist with 20 years of experience.

### 2.2. MRI Protocol

MRI examinations were performed using a single 1.5 T scanner (Achieva dStream, Philips Medical Systems, Best, The Netherlands) with a balloon-covered expandable endorectal coil (BPX-15™, Bayer Medical Care, Indianola, PA, USA) paired with a phased array 32-channel surface coil. Gastrointestinal peristalsis was suppressed by intramuscular administration of 20 mg of scopolamine-butylbromide. Detailed mpMRI imaging protocol is reported in Table 1. Data from DCE-MRI (intravenous bolus injection of 0.1 mmol/kg of gadobutrol) were acquired for clinical needs but not considered for quantitative analysis.

### 2.3. MRI Qualitative Analysis 

Two dedicated radiologists (5 and 4 years of experience in prostate mpMRI) independently reviewed the images and identified the index lesion based on PIRADS v. 2.0 classification. Images were than compared with prostatectomy reports to check the concordance between imaging and pathology findings. In all cases, the index lesion at MRI matched the lesion with highest GGG at pathology. In case of multiple lesions at MRI, with same GGG at pathology, the largest one in the images was selected for the analysis.

### 2.4. MRI Quantitative Analysis

#### 2.4.1. MRI Images Post-Processing

T2w images were pre-processed to account for the impact of bias field and intensity non-standardness on T2w images [11], by N4 normalization [12] and linear scaling and shifting within 0–600 in 3D Slicer v. 4.10.2 [13].

Diffusion weighted images were processed using monoexponential model with *b*-values = 50 s/mm^2^ and *b*-values = 800 s/mm^2^, to obtain ADC maps.

#### 2.4.2. Tumor Volume Segmentation

**Figure 1:**
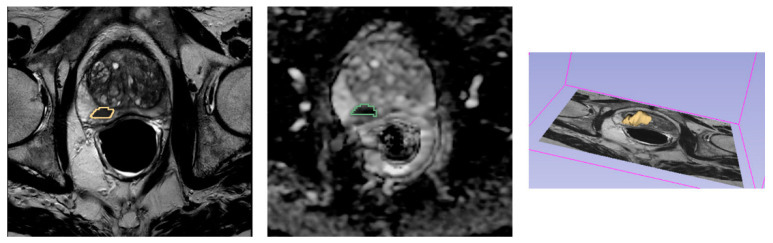
Example of an index lesion segmentation on T2w image (**left**) and ADC map (**center**) and the resulted 3D segmented volume of interest (VOI) (**right**).

Index lesions were segmented both on T2w images and ADC maps using a semi-automatic approach based on region growing [14], as implemented in 3D Slicer v. 4.10.2 [13], and the 3D lesion volume (volume of interest (VOI)) was obtained. In order to avoid registration errors as well as motion artifacts, tumors were segmented separately on T2w images and ADC maps (see Figure 1).

As a preliminary step, to evaluate the stability of the quantitative imaging features with respect to segmentation, two radiologists independently performed tumor delineation. For subsequent analysis in a separate session, the two readers re-segmented in consensus the images of the entire patient sample.

Only patients with segmented VOI greater 0.7 cc in ADC were included in the subsequent analysis. Dimensional cut-off on images were empirically evaluated, considering that a lesion with less than 4 voxels in each direction would not make for meaningful calculation of different textural parameters [6].

#### 2.4.3. MRI Image Quantification

Feature extraction was performed by using Radiomics module of 3D slicer v. 4.10.2 [15]. Segmented VOIs were resampled to isotropic voxel spacing, using an upsampling scheme based image slice thickness. Image re-segmentation was performed with Collewet normalization [16] and VOI intensities were discretized to a fixed number of 64 bins.

A total number of 93 imaging features (IFs) was extracted from both T2w images (T2w-IFs) and ADC map (ADC-IFs), without applying filters, from five different classes (14 morphological features (M), 18 first-order statistical features from intensity histogram (FOS), including mean ADC value, and 61 textural features from analysis of Gray Level Co-occurrence Matrix (GLCM), Gray Level Size Zone Matrix (GLSZM), Gray Level Run Length Matrix (GLRLM), and Neighborhood Grey Tone Difference Matrix (NGTDM). Details on radiomic pipeline for feature extraction can be found in Table S1 of Supplementary Materials.

To evaluate the impact of semi-automatic segmentation on image quantification, the nonparametric repeated measurement Friedman test was used to calculate a stability rank per feature from independent segmentations performed by two readers [17,18]. Furthermore, feature selection was carried out on the extracted IFs for each image modality in order to rule out redundant features, by analyzing the covariance matrix. Clusters of highly correlated features (r > 0.8) were reduced to a single representative imaging feature, using the one presenting the highest inter-subject’s range [6], calculated by Coefficient of Variation [17,18]. For subsequent analysis, only non-redundant IFs, on the basis of the stability rank for delineation inaccuracies by different operators, were retained.

#### 2.4.4. Association of Radiomic Phenotype and Clinical Data

Unsupervised hierarchical clustering analysis was performed to group patients on the basis of similar radiomic patterns. Each patient was considered a statistical unit (sample) characterized by different measurable properties (IFs values of patient’s index lesion). The clustering procedure grouped the patients showing a closer radiomic pattern, as defined by a distance criterion (Euclidean distance).

Before the clustering analysis, IFs presenting extreme values were log2 transformed, replacing zero values with the smallest positive value of the radiomic phenotype, over all patient samples, before transformation. IFs were standardized to zero mean and a unit standard deviation. Heatmaps were automatically generated by using ComplexHeatmap in R-package [19].

Fisher’s exact test as implemented was used to test statistical significance of the association between identified clusters and patient histological characteristics.

#### 2.4.5. Building Diagnostic Radiomic Signature

Different signatures composed by IFs from T2w images and ADC maps independently and by IFs from the two modality jointly were built and tested for prediction of high (GGG ≥ 4 + 3) vs. low (GGG < 4 + 3) GGG, presence vs. absence of ECE, and pN status (pN0 vs. pN ≥ 1). For each image modality we selected only non-redundant IFs within the first 10 stable Ifs in the stability rank (S_TOP_). ADC_mean_ was evaluated both separately and in combination with Ifs signatures, to test the effective potential advantage of textural features compared to standard value ADC_mean_. A Support Vector Machine model was implemented using a R-package [20,21] with kernel radial in order to evaluate the combination of features that achieved the best performances in the prediction model. Classification performances were evaluated in terms of accuracy, sensitivity, and specificity as metrics. Since an imbalance exists between the different classes to be considered in the classification, we employed data resampling, with an undersampling method [22,23]. We randomly resampled the cohort, removing samples from the majority class in order to obtain 10 random subsets with the same number of samples in the two classes. Then we performed the classification in each of these subsets, so the classification was performed 10 times. The overall mean value of accuracy, specificity, and sensitivity was obtained as means over the 10 repetitions.

Since the biology upon peripheral and transition zone lesions could be different across characteristics, the classification was performed also in the subset of PZ lesions, in order to evaluate the performance of signatures in this subgroup. Considering the low number of TZ lesions, no classification was made on these samples.

## 3. Results

From the initial population of 102 patients, 40 of them were excluded according to the imaging volumetric criteria (VOI < 0.7 cc in ADC map). The final population included for the analysis consisted of 62 patients (age range 44–79 years) with 62 index lesions.

Lesions characteristics at MRI and pathological analysis are reported in Table 2.

On a subset of patients, the index lesion was segmented by two operators, and a stability rank with respect to segmentation was defined for all the 93 IFs extracted from both T2w images and ADC maps. Forty-four out of ninety-three T2w-IFs and 61/93 ADC-IFs resulted in being stable with respect to semi-automatic segmentation (*p*-value of Friedman test > 0.05). Among them, the analysis of covariance matrix allowed to select 17 T2w-IFs and 23 ADC-IFs as not-redundant futures used for further multivariate analysis.

**Figure 2:**
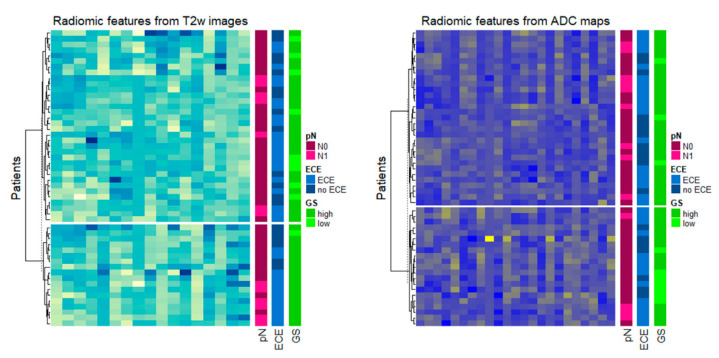
Radiomic patterns obtained on T2w images (**left**) and ADC maps (**right**); the dendrogram at the left side represents the patient’ grouping obtained from the clustering procedure; annotation at the right side represent the distribution of Gleason Grade Group (GGG), extracapsular extension (ECE), and nodal stage (pN) status in the groups.

**Figure 3:**
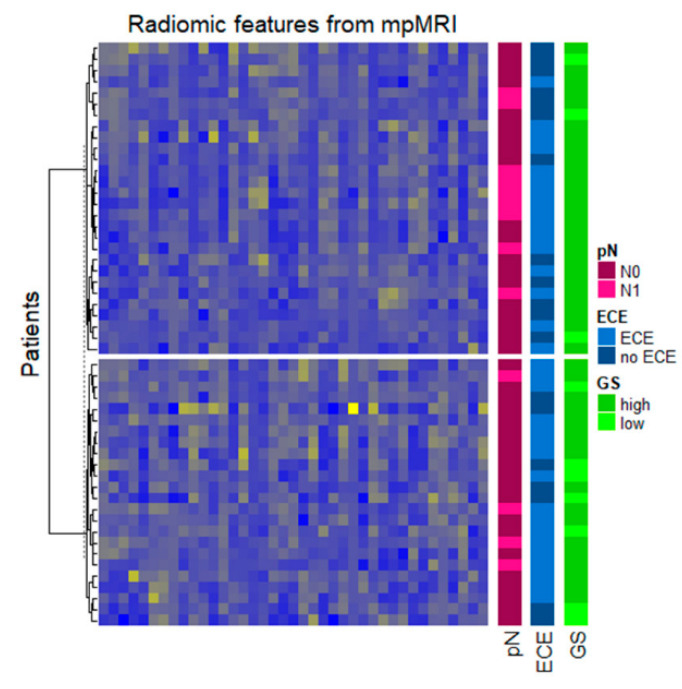
Radiomic patterns obtained considering both T2w images and ADC maps; the dendrogram at the left side represents the patient’ grouping obtained from the clustering procedure; annotation at the right side represent the distribution of GGG, ECE, and pN status in the groups.

Unsupervised hierarchical clustering revealed two major groups of patients both on the basis of T2w radiomic pattern as well as on the basis of ADC radiomic pattern (Figure 2) or considering IFs from both T2w images and ADC maps (Figure 3).

T2w radiomic pattern was found to be associated with pN, ECE, and GGG (*p*-value: 0.027, 0.05, and 0.03, Fisher’s exact test), while the ADC maps’ radiomic pattern was found to be associated with GGG (*p*-value: 0.04, Fisher’s exact test).

When combining imaging biomarkers from T2w images and ADC maps, the multimodal radiomic patterns of the two patient groups identified by unsupervised hierarchical clustering analysis was found to be associated with GGG (*p*-value: 0.05 Fisher’s exact test). To build and test a diagnostic radiomic signature, we focused the analysis on a reduced set of stable and not-redundant imaging features. Table 3 shows different signature models, obtained from T2w images and ADC maps independently and from the two modalities jointly with and without the inclusion of ADC_mean_, together with models’ performance (mean, max, min, and standard deviation of performances obtained by data resampling) in predicting GGG, ECE, and nodal involvement. 

**Figure 4:**
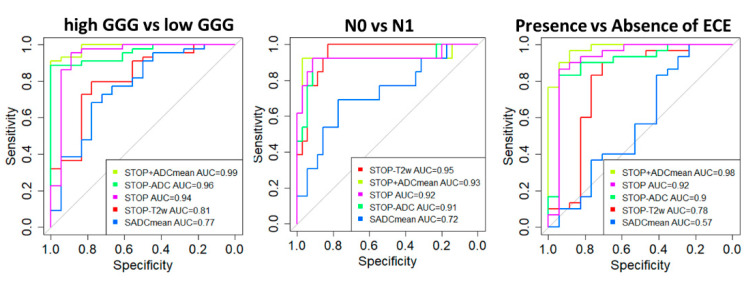
ROC curves and AUC values for classification.

Figure 4 shows ROC curves and AUC for classification.

The best performance was reached by the signature combing IFs from T2w images and ADC map (S_TOP-T2w/ADC)_ with an accuracy in the prediction of GGG, nodal involvement, and ECE of 0.88, 0.9m, and 0.85, respectively. The performance was even improved when ADC_mean_ was added to the model (accuracy: 0.90, 0.89, 0.88). Considering each single signature, similar performances were obtained for the dataset of PZ lesions, except for S_ADCmean_, which increases significantly, confirming the predictive role of DWI in PZ lesions (see Table S2 and Figure S1 in Supplementary Materials). Despite this, a slightly higher accuracy has been found for the multimodal signature S_TOP_ (see Table S2 in Supplementary Materials).

## 4. Discussion

Due to the wide spectrum of clinical presentation and possible outcome of PCa cancer, identifying the optimal treatment option for patients represents the most challenging problem in PCa clinical workup. There is a growing attention in finding reliable tools that could provide a non-invasive assessment of PCa aggressiveness, allowing for a patient-tailored management, ranging from radical-prostatectomy with nodes dissection to active surveillance. Radiomics can analyze a large number of imaging features that cannot be visualized by radiologists, and its potential role in the implementation of tumor detection, characterization, and treatment response has been explored in several oncological settings [24,25,26]. A large variability of methodological approaches exists among previous studies exploring the application of radiomics to prostate mpMRI. Moreover, the analysis are usually based on single MR quantitative parameters while a multiparametric signature would be preferred, reflecting the multiparametric approach of qualitative clinical reporting [8,9]. From a methodological point of view, our pipeline was designed and standardized in order to identify accurate and reproducible imaging biomarkers addressing the different issues related to the extraction of high-throughput quantitative biomarkers, as underlined by IBSI initiative [27].

The first issue was represented by lesion volume definition. Even if advanced approaches were developed in recent years for PCa segmentation [28], in PCa, as in other oncological diseases, manually contouring is the standard method to define lesion volume [26,27,28], but, due to its dependence from operator expertise [6], it has been proved to be suboptimal in radiomic studies. In different studies, e.g., [29], lesion volume is defined on a single image slice, thus limiting the fully characterization of volumetric extension of lesions. In order to overcome these limitations, we used a semi-automatic segmentation approach based on region growing on T2w images and ADC maps separately, followed by manual adjustment, as suggested by IBSI.

In order to warrant the reproducibility of IFs included in the radiomic signature, we evaluated a rank of inter-observer stability for IFs extracted from tumor volumes from both T2w images and ADC maps [6,17]. The subsequent analysis was then performed on a set of robust and reproducible IFs. Furthermore, the use of a single MR scanner using a standardized acquisition protocol for all involved patients allowed to avoid issues related to IFs dependency from scanner and acquisition protocols.

Several studies described the association of radiomic features of T2w and DWI images with GGG, but there is a large heterogeneity both in the preselection of quantitative feature to test and in the results. In their study, Nketiah et al. [29] selected 4 distinct textural features extracted from T2w from the 14 GLCM textural features originally proposed by Haralick. They found that among the T2W image textural features ASM and entropy correlated significantly (*p* < 0.05) with GGG. In the study of Wibner et al. [8], higher PZ GGG cancer was associated with higher Entropy and lower Energy extracted from ADC maps. None of the texture features of T2w images showed significant associations with GGG. According to the authors, to effectively predict PCa aggressiveness, the identification of an IF signature could be more effective than the identification of specific and isolated features.

Toivonen et al. [30] have recently proposed a combination of features, selected from a pool of 7105 IFs extracted from T2w, DWI a T2 mapping, in a cohort of patients comparable to ours, showing an AUC of 0.88 for the classification of low vs. high GGG. Considering our cohort dimension and the need of an imaging post-processing methodologies applicable in everyday clinical practice, we aimed to limit the analysis to a reasonable number of accurate and reproducible features. For this reason, in this work, a signature composed of the top 10 stable and non-redundant features was tested.

We also attempted to implement a quantitative model able to predict alongside GGG also ECE and nodal involvement. Knowledge of ECE at the time of diagnosis can affect management decisions, with a potential impact on treatment modality or surgical technique. The preoperative prediction of nodal involvement is even more important for clinical practice and it is currently based on clinical and pathological data [31]. As a matter of fact, even though some qualitative MRI parameters, such as dimension and length of capsular contact of dominant lesion, have been identified as a predictor of ECE and nodal involvement [32], due to its subjective nature, the evaluation of these parameters is plagued by low inter-observer agreement [33], and a high number of cases still suffer from over diagnosis and overtreatment.

Using a limited combination of IFs from T2w and ADC images, selected among the top 10 more stable features, we were able to predict GGG, ECE, and node status with a sensitivity greater than 0.88 and a specificity of 0.78 at least (Table 3).

Since mean ADC value in tumor volumes is known to be a quite reliable biomarker for tumour aggressiveness [34,35], we tested the effective potential advantage of radiomic IFs with respect to the easier use of ADC_mean_. Interestingly, mean ADC alone showed a lower accuracy with respect to the use of radiomic IFs, but when used in combination (ADC mean + S_TOP-T2/ADC_), an improvement in model performance was obtained, particularly for prediction of GGG and ECE (Table 3).

In this study, no information such as genomic and proteomic data of the tumors was included since this information is not usually evaluated in routine clinical practice. However, it would be interesting, in prospective works, to include other biological parameters possibly underlying molecular changes of PC that may reflect in radiomic features (e.g., mRNAs or miRNAs expression levels). Such molecular features could help to decipher the biological role of radiomic features in capturing genotype in its living environment and eventually complete and improve the radiomic signature to predict clinical endpoints.

This study has some limitations most related to the small, even though homogeneous, sample size. Indeed, our inclusion criteria required that enrolled patients performed both mpMRI and image-guided biopsy, thus obtaining a cohort of biopsy-confirmed patients to be analyzed by radiomic mpMRI. Then, patients received prostatectomy as primary treatment, which is nowadays recommended only for patients showing high-grade tumors since can be postponed for localized, low-grade (Gleason < 7) disease without significant change in outcome [36]. Moreover, we also considered a subset of patients undergoing lymphadenectomy during surgery in order to assess the predictiveness of mpMRi-based signatures with respect to lymph node status. Furthermore, in order to warrant rigorous radiomic analysis, we had to exclude, from our enrolled cohort, those patients with lesion dimensions on mMRI below 0.7 cc. We were very conservative on this point to warrant an accurate and meaningful radiomic analysis. This cut-off for lesion volume was due to the lower spatial resolution of ADC maps that were included in our multiparametric signature. In the majority of published radiomics studies on PCa, tumors were analyzed on the basis of their dimensions as evaluated by histology (0.5 cc dimensional cut-off for clinically significant tumors) [8,29,37]. Despite this, a radiomic analysis on less than 4 image voxels in each direction make no meaningful the calculation for different textural parameters [6]. For those reasons, we were not able to perform an external validation of our radiomic signatures without further impacting on samples to be used for training and internal validation.

Limitation in terms of VOI dimensions is clearly an issue of radiomics [6], which could have an impact from a clinical point of view. Despite this, in our cohort with the application of a dimensional cut off of 0.7 cc, the large majority of discarded patients have small PCas on imaging characterized by a low/intermediate aggressiveness at pathology (53% were GGG 3 + 4). As pointed out, the dimensional cut-off was defined considering the lower spatial resolution of ADC maps for our multiparametric signature. However, due to higher resolution of T2w images, the minimum VOI volume was required for radiomic analysis of T2w images. Thus, mono-modal T2w signature could be used to evaluate aggressiveness in case of small PCas.

Even if we did not separate PZ and TZ lesions for the analysis due to the disproportion in frequencies distribution, with PZ tumors accounting for almost 70% of lesions, performances of the different signatures in the subset of PZ lesions have been evaluated. The obtained results confirmed the predictive role of DWI in PZ lesions, even if the multivariate signature S_TOP_ has shown slightly higher accuracy. Despite this, a larger cohort of TZ lesions will allow to confirm the specific impact of signature in this subset of PC patients. Finally, we focused our investigation on radiomic features extracted from T2w images and ADC map, excluding data from DCE imaging. Although our approach could be considered in agreement with PIRADS guidelines, which consider DCE only as a support to T2 and DWI sequences for lesion detection and characterization, it could be interesting to investigate the additional contribution of radiomic features from DCE.

In conclusion, our data suggest that the implementation of robust radiomic-pipeline for the analysis of mpMRI allows for the extraction of a reliable radiomic signature, potentially able to predict PCa aggressiveness, in terms of Gleason score, extracapsular extension, and nodal stage.

## Figures and Tables

**Table 1:** Imaging protocol details.

Parameter	T2 TSE * Axial	T2 TSE * Sagittal	T2 TSE * Coronal	DWI ** (b: 0, 800, 1600)	DCE ***
TR (ms)	4824	4370	2991	4376	3.7
TE (ms)	120	120	120	80	1.83
FOV° (mm)	180 × 180	180 × 180	180 × 180	180 × 180	180 × 180
Matrix Thickness (mm)	3	3	3	3	3
Gap (mm)	0.3	0.3	0.3	0.3	0
Flip angle (°)	90	90	90	90	8/5, 8, 12, 15
Acquisition time	4 min 6 s	3 min 25 s	2 min 8 s	5 min 19 s	3 min 20 s

* TSE: Turbo spin echo imaging; ** DWI: Diffusion-weighted imaging; *** DCE: Dynamic contrast-enhanced imaging; ° FOV: Field of view.

**Table 2:** Index lesions imaging and pathological characteristics.

		^#^ Patients	Frequency
Index lesion location	PZ *	43	69%
	TZ **	16	26%
	Both	3	5%
PI-RADS ***	3	3	5%
	4	30	48%
	5	29	47%
Gleason Score	7 (3 + 4)	18	29%
	7 (4 + 3)	18	29%
	8	5	8%
	9	21	34%
ECE °	Yes	38	61%
	No	24	39%
pN	pN0 °°	39	63%
	pN1 ≥ 1 °°°	13	21%
	pNx ^#^	10	16%

* PZ: Peripheral Zone; ** TZ: Transition Zone; *** PI-RADS: Prostate Imaging-Reporting and Data System; ° ECE: Extracapsular extension; °° pN0: absence of nodal metastases at pathologic examination, °°° pN1: presence of nodal metastases at pathologic examination. ^#^ pNx: nodes status not assessable.

**Table 3:** Signature models and performances. Different signature models obtained from T2w images and ADC maps independently and from the two modalities jointly and the corresponding diagnostic performances. On the left, the specific imaging features (IFs) included in each model are reported. Accuracy, sensitivity, and specificity are reported as Mean and Standard Deviation, while in parenthesis, minimum and maximum over 10 repetitions are reported.

Signature	Image Modality	Feature Group	Features	High GGG vs. Low GGG			N0 vs. N1			Presence vs. Absence of ECE		
				Acc ^#^	Sens ^##^	Spec ^###^	Acc ^#^	Sens ^##^	Spec ^###^	Acc ^#^	Sens ^##^	Spec ^###^
S_TOP-T2w_ *	T2w images	M	Sphericity	0.75 ± 0.05 (0.67–0.83)	0.73 ± 0.11 (0.56–0.89)	0.76 ± 0.14 (0.44–0.94)	0.84 ± 0.05 (0.77–0.92)	0.79 ± 0.09 (0.62–0.92)	0.88 ± 0.09 (0.69–1)	0.75 ± 0.03 (0.71–0.79)	0.8 ± 0.07 (0.76–0.94)	0.69 ± 0.12 (0.47–0.94)
		GLRLM	SRHGLE									
		GLSZM	SAHGLE									
			LAHGLE									
S_TOP-ADC_ **	ADC maps	M	Elongation	0.83 ± 0.06 (0.75–0.94)	0.95 ± 0.04 (0.89–1)	0.7 ± 0.13 (0.5–0.89)	0.86 ± 0.05 (0.73–0.92)	0.77 ± 0.1 (0.62–0.92)	0.95 ± 0.09 (0.77–1)	0.81 ± 0.02 (0.79–0.85)	0.85 ± 0.1 (0.65–1)	0.78 ± 0.09 (0.59–0.94)
			Flatness									
		GLCM	Inverse Variance									
			Cluster Shade									
		NGTDM	Busyness									
S_TOP_ ***	T2w images	M	Sphericity	0.88 ± 0.04 (0.81–0.94)	0.94 ± 0.04 (0.89–1)	0.82 ± 0.1 (0.67–0.89)	0.9 ± 0.04 (0.85–0.96)	0.9 ± 0.1 (0.69–1)	0.9 ± 0.1 (0.69–1)	0.85 ± 0.04 (0.79–0.91)	0.93 ± 0.06 (0.88–1)	0.78± 0.09 (0.65–0.94)
		GLRLM	SRHGLE									
		GLSZM	SAHGLE									
			LAHGLE									
	ADC aps	M	Elongation									
			Flatness									
		GLCM	Inverse Variance									
			Cluster Shade									
		NGTDM	Busyness									
S_ADCmean_ °	ADC maps	-	ADC_mean_	0.71 ± 0.04 (0.64–0.78)	0.64 ± 0.1 (0.44–0.78)	0.78 ± 0.05 (0.72–0.83)	0.67 ± 0.06 (0.54–0.73)	0.51 ± 0.15 (0.23–0.77)	0.82 ± 0.14 (0.62–1)	0.63 ± 0.03 (0.59–0.68)	0.49 ± 0.16 (0.29–0.76)	0.76 ± 0.19 (0.41–1)
S_TOP + ADC mean_ °°	T2w images	M	Sphericity	0.9 ± 0.04 (0.83–0.94)	0.93 ± 0.06 (0.83–1)	0.88 ± 0.06 (0.78–0.94)	0.89 ± 0.04 (0.81–0.92)	0.82 ± 0.11 (0.62–1)	0.96 ± 0.06 (0.85–1)	0.88 ± 0.03 (0.82–0.91)	0.88 ± 0.07 (0.71–1)	0.89 ± 0.07 (0.76–1)
		GLRLM	SRHGLE									
		GLSZM	SAHGLE									
			LAHGLE									
	ADC maps	M	Elongation									
			Flatness									
		GLCM	Inverse Variance									
			Cluster Shade									
		NGTDM	Busyness									
		-	ADC_mean_									

* S_TOP-T2w_: S_TOP_ signature extracted from T2w images; ** S_TOP-ADC_: S_TOP_ signature extracted from ADC maps; *** S_TOP_: S_TOP_ signature extracted from both T2w images and ADC maps; ° S_ADCmean_: signature based on ADC_mean_; °° S_TOP + ADCmean_: signature obtained by combining S_TOP_ and ADC_mean_; ^#^ Acc: accuracy; ^##^ Sens: sensitivity; ^###^ Spec: specificity.

## Data Availability

Data sharing not applicable.

## Supplementary Materials

https://www.mdpi.com/article/10.3390/diagnostics11040594/s1, Figure S1: ROC curves and AUC values for classification in PZ patient dataset. Table S1: Report on image processing and image biomarker extraction, Table S2: Signature models and performances for classification of PZ lesions.
