# Supplementary material for: Advanced Imaging Analysis in Prostate MRI: Building a Radiomic Signature to Predict Tumor Aggressiveness
Source: Diagnostics (Basel). 2021 Mar 26;11(4):594. doi: 10.3390/diagnostics11040594 (PMC8065545; doi:10.3390/diagnostics11040594)
Supplement: Supplementary file 1 [file diagnostics-11-00594-s001.pdf]

Supplemental Table 1. Report on image processing and image biomarker extraction.

Topic	Modality	Item	Description
Image modality			MRI: T2w and DWI acquisitions
In-plane resolution		28	T2w images: 0,35 mm x 0,35 mm<br>DWI images:1,125 mm x 1,125 mm
Image slice thickness		29	T2w images: 3,3 mm<br>DWI images: 3,3 mm
Diffusion-weigh ted imaging	DWI-MRI	36	b- values = 50 s/mm2 and 800 s/mm2
ADC computation	DWI-MRI	39	Monoexponential model (1)
Non-uniformity correction	MRI	45	N4 normalisation (2)
Intensity normalisation	MRI	46	Normalisation to to fixed intensity range (3)
Segmentation method		48a	Semi-automatic VOI definition followed by manual adjustment
		48b	Two radiologists
		48c	FastGrowCut (4), based on region growing
		48d	Segmentations performed separately on T2w images and ADC maps
Re-segmentation methods		55	Resegmentation based on Collewet normalization (5)
Discretisation method		56a	Fixed Bin Count (FBN)
		56b	64

Supplemental Table 2: Signature models and performances for classification of PZ lesions. Different signature models obtained from T2w images and ADC maps independently and from the two modalities jointly and the corresponding diagnostic performances. On the left are reported the specific IFs included in each model. Accuracy, sensitivity and specificity are reported as Mean and Standard Deviation, while in parenthesis are reported minimum and maximus of performances

Signature name	Image modality	Feature group	Features	high GGG vs low GGG			N0 vs N1			Presence vs Absence of ECE		
$S_{TOP-T2w}$*	T2w images	M	Sphericity	***Acc***[#]	***Sens***[##]	***Spec***[###]	***Acc***[#]	***Sens***[##]	***Spec***[###]	***Acc***[#]	***Sens***[##]	***Spec***[###]
		GLRLM	SRHGLE	0,76 ±0,04<br>(0,71 -0,83)	0,83 ±0,12<br>(0,67 -1)	0,7 ±0,13<br>(0,58 -1)	0,86 ±0,09<br>(0,65 -0,95)	0,8 ±0,12<br>(0,6 -0,9)	0,92 ±0,1<br>(0,7 -1)	0,8 ±0,08<br>(0,65 -0,9)	0,97 ±0,05<br>(0,9 -1)	0,62 ±0,15<br>(0,3 -0,8)
		GLSZM	SAHGLE<br>LAHGLE									
$S_{TOP-ADC}$**	ADC maps	M	Elongation<br>Flatness	0,91 ±0,04<br>(0,83 -0,96)	0,98 ±0,03<br>(0,92 -1)	0,83 ±0,08<br>(0,67 -0,92)	0,92 ±0,04<br>(0,85 -1)	0,84 ±0,08<br>(0,7 -1)	1 ±0<br>(1 -1)	0,84 ±0,08<br>(0,7 -0,95)	0,78 ±0,13<br>(0,5 -1)	0,89 ±0,07<br>(0,8 -1)

		GLCM	Inverse Variance<br>Cluster Shade									
		NGTDM	Busyness									
S_{TOP}***	T2w images	M	Sphericity	0,92 ± 0,04 (0,88-0,96)	0,98 ± 0,03 (0,92-1)	0,85 ± 0,1 (0,75-1)	0,95 ± 0,04 (0,9-1)	0,89 ± 0,07 (0,8-1)	1 ± 0 (1-1)	0,9 ± 0,04 (0,85-0,95)	0,87 ± 0,11 (0,7-1)	0,92 ± 0,09 (0,7-1)
		GLRLM	SRHGLE									
		GLSZM	SAHGLE<br>LAHGLE									
	ADC maps	M	Elongation<br>Flatness									
		GLCM	Inverse Variance<br>Cluster Shade									
		NGTDM	Busyness									
$S_{ADCmean}$ °	ADC maps	-	ADC_{mean}	0,91 ± 0,05 (0,83-0,96)	0,92 ± 0,09 (0,75-1)	0,89 ± 0,15 (0,67-1)	0,94 ± 0,04 (0,9-1)	0,92 ± 0,09 (0,8-1)	0,96 ± 0,07 (0,8-1)	0,68 ± 0,05 (0,6-0,75)	0,71 ± 0,14 (0,5-0,9)	0,64 ± 0,13 (0,4-)
$S_{TOP + ADC mean}$ °°	T2w images	M	Sphericity	0,85 ± 0,05 (0,79-0,92)	0,81 ± 0,09 (0,67-0,92)	0,89 ± 0,09 (0,75-1)	0,86 ± 0,05 (0,8-0,95)	0,75 ± 0,12 (0,6-1)	0,96 ± 0,05 (0,9-1)	0,91 ± 0,05 (0,8-0,95)	0,9 ± 0,12 (0,6-1)	0,92 ± 0,06 (0,8-1)
		GLRLM	SRHGLE									
		GLSZM	SAHGLE<br>LAHGLE									
	ADC maps	M	Elongation<br>Flatness									
		GLCM	Inverse Variance<br>Cluster Shade									
		NGTDM	Busyness									
	ADC maps	-	ADC_{mean}									

*$S_{TOP-T2w}$: S_{TOP} signature extracted from T2w images; **$S_{TOP-ADC}$: S_{TOP} signature extracted from ADC maps; ***S_{TOP} : S_{TOP} signature extracted from both T2w images and ADC maps; °$S_{ADCmean}$: signature based on ADC_{mean}; °°$S_{TOP+ADCmean}$: signature obtained by combining °°°$S_{TOP-T2w/ADC}$ and ADC_{mean}; #*Acc*: accuracy; ##*Sens*: sensitivity; ###*Spec*: specificity

Supplemental Figure S1: ROC curves and AUC values for classification in PZ patient dataset

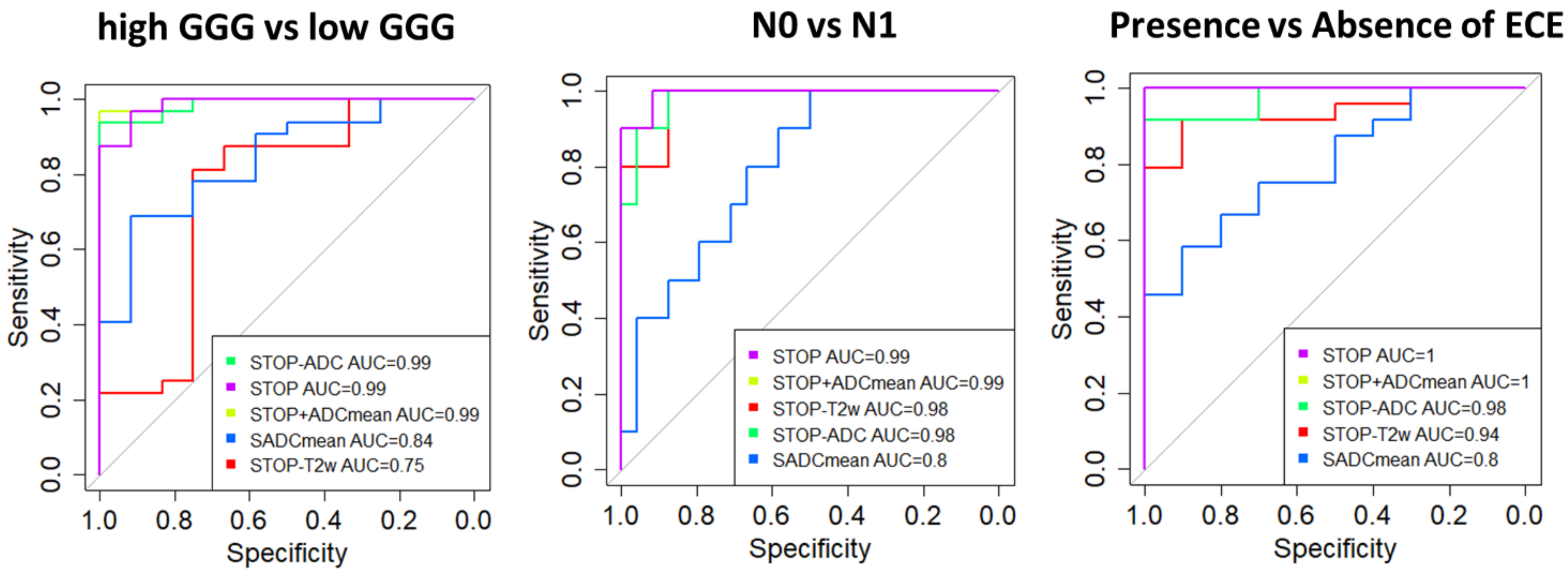

References

1. Partridge SC, Nissan N, Rahbar H, Kitsch AE, Sigmund EE. Diffusion-weighted breast MRI: Clinical applications and emerging techniques. J Magn Reson Imaging 2017;45(2):337-355.
2. Tustison NJ, Avants BB, Cook PA, et al. N4ITK: improved N3 bias correction. IEEE Trans Med Imaging 2010;29(6):1310-1320.
3. Palumbo D, Yee B, O'Dea P, Leedy S, Viswanath S, Madabhushi A. Interplay between bias field correction, intensity standardization, and noise filtering for T2-weighted MRI. IEEE Eng Med Biol Soc. Volume 2011; 2011. p. 5080-5083.

4. Liangija Zhu IK, Yi Gao, Ron Kikinis, Allen Tannenbaum. An Effective Interactive Medical Image Segmentation Method using Fast GrowCut. Int Conf Med Image Comput Comput Assist Interv Workshop on Interactive Methods (MICCAI); 2014.

5. Collewet G, Strzelecki M, Mariette F. Influence of MRI acquisition protocols and image intensity normalization methods on texture classification. Magn Reson Imaging 2004;22(1):81-91.
